# MiR-380 inhibits the proliferation and invasion of cholangiocarcinoma cells by silencing LIS1

**DOI:** 10.1186/s12935-024-03241-4

**Published:** 2024-04-06

**Authors:** Zhicheng Wei, Bowen Xu, Yanjiang Yin, Jianping Chang, Zhiyu Li, Yefan Zhang, Xu Che, Xinyu Bi

**Affiliations:** 1 Department of Hepatobiliary Surgery, National Cancer Center/National Clinical Research Center for Cancer/Cancer Hospital, Chinese Academy of Medical Sciences and Peking Union Medical College, Beijing, 100021 China; 2 Department of Hepatobiliary and Pancreatic Surgery, National Cancer Center/National Clinical Research Center for Cancer/Cancer Hospital & Shenzhen Hospital, Chinese Academy of Medical Sciences and Peking Union Medical College, Shenzhen, 518116 China; 3 Key Laboratory of Gene Editing Screening and Research and Development (R&D) of Digestive System Tumor Drugs, National Cancer Center/National Clinical Research Center for Cancer/Cancer Hospital, Chinese Academy of Medical Sciences and Peking Union Medical College, Beijing, 100021, China

**Keywords:** Cholangiocarcinoma, miR-380, LIS1

## Abstract

**Background:**

The objective of this study was to determine the role and regulatory mechanism of miR-380 in cholangiocarcinoma.

**Methods:**

The TargetScan database and a dual-luciferase reporter assay system were used to determine if LIS1 was a target gene of miR-380. The Cell Counting Kit 8 assay, flow cytometry, and Transwell assay were used to detect the effects of miR-380 and LIS1 on the proliferation, S-phase ratio, and invasiveness of HCCC-9810/HuCCT1/QBC939 cells. Western blotting was used to determine the effect of miR-380 on MMP-2/p-AKT. Immunohistochemistry detected the regulatory effect of miR-380 on the expression of MMP-2/p-AKT/LIS1.

**Results:**

Expression of miR-380 in cholangiocarcinoma was decreased but expression of LIS1 was increased. LIS1 was confirmed to be a target gene of miR-380. Transfection with miR-380 mimics inhibited the proliferation, S-phase arrest, and invasion of HCCC-9810/HuCCT1/QBC939 cells, and LIS1 reversed these inhibitory effects. miR-380 inhibitor promoted proliferation, S-phase ratio, and invasiveness of HCCC-9810/HuCCT1/QBC939 cells. si-LIS1 salvaged the promotive effect of miR-380 inhibitor. Overexpression of miR-380 inhibited expression of MMP-2/p-AKT/LIS1, but miR-380 inhibitor promoted their expression.

**Conclusion:**

An imbalance of miR-380 expression is closely related to cholangiocarcinoma, and overexpression of miR-380 inhibits the expression of MMP-2/p-AKT by directly targeting LIS1.

**Supplementary Information:**

The online version contains supplementary material available at 10.1186/s12935-024-03241-4.

## Background

Primary liver cancer is one of the most common malignancies worldwide [1, 2]. It is the fourth most common malignant tumor and the second cause of cancer in China [3, 4]. Primary liver cancer includes three different pathological types: hepatocellular carcinoma, intrahepatic cholangiocarcinoma, and combined hepatocellular carcinoma–cholangiocarcinoma. The three types differ greatly in pathogenesis, biological behavior, histopathology, response to treatment, and prognosis, and HCC accounts for 75–85% of primary liver cancers and intrahepatic cholangiocarcinoma accounts for 10–15% [5].

Cholangiocarcinoma originates from bile duct epithelium, and was once considered a rare tumor [6, 7]. Cholangiocarcinoma is a general term used to describe hilar, distal and intrahepatic cholangiocarcinoma, and it is one of the most difficult diseases of the biliary system to treat [8, 9]. A recent study has indicated that the incidence of cholangiocarcinoma is increasing, and the prognosis is poor [8]. The 5-year survival rate of intrahepatic cholangiocarcinoma is 2–15% and that of extrahepatic cholangiocarcinoma is 2–30% [10, 11].

Although the etiology of cholangiocarcinoma has not been determined, several risk factors have been identified. Risk factors for intrahepatic cholangiocarcinoma include primary sclerosing cholangitis, cirrhosis, and parasitic infection, while primary sclerosing cholangitis and bile duct stones are risk factors for extrahepatic cholangiocarcinoma [12, 13]. Surgery is the most effective and preferred treatment for cholangiocarcinoma [14]. However, the tumor can only be removed completely in about 35% of patients with early cholangiocarcinoma [15]. The postoperative recurrence rate is high even in patients with complete resection [16, 17]. Cholangiocarcinoma commonly develops multi-resistance to chemotherapeutic drugs because of the unique location of the tumor, and is prone to invasion of surrounding tissues, blood vessels and nerves, and distant metastasis in the early stage [18–20].

Approximately 60–70% of newly diagnosed cases of cholangiocarcinoma are at an advanced stage and are only candidates for palliative treatment, including chemotherapy [21, 22]. The molecular mechanism by which cholangiocarcinoma develops and progresses has not been determined. Understanding the molecular mechanisms of cholangiocarcinoma may help to develop methods of early diagnosis and novel treatments.

Lissencephaly 1 (LIS1) is located in the 17p13.3 human chromosome region, and was the first cloned gene that was identified as being related to a brain neuron migration disorder [23]. LIS1 is involved in cell proliferation, migration and transport, and is related to the pathogenesis of cholangiocarcinoma [24]. However, LIS1 is also abnormally expressed in a variety of tumors and thus is not useful for early screening for cholangiocarcinoma [25, 26]. miRNAs are a class of noncoding RNAs that are about 20 nucleotides in length. An important function of miRNAs is regulation of transcription, and many have a regulatory function in a variety of malignancies. Expression of several miRNAs is altered in cholangiocarcinoma [27]. For example, expression of miR-19b-3p is significantly increased in cholangiocarcinoma tissue, and promotes proliferation of cholangiocarcinoma and epithelial mesenchymal transformation [28]. miR-129-2-3p inhibits the proliferation and invasion of cholangiocarcinoma by directly targeting the *Wip1* gene [29]. In our preliminary research, PITA, miRmap, and microT database screening identified *LIS1* as a potential target gene of miR-380. However, the role of miR-380 in cholangiocarcinoma has not been investigated.

Thus, the aims of this study were to determine the effects of miR-380 on the proliferation, S-phase arrest, and invasiveness of cholangiocarcinoma cells, identify key regulatory target genes of miR-380, and clarify the role and mechanism of miR-380 in cholangiocarcinoma.

## Methods

### Patients

The study included 36 patients with cholangiocarcinoma diagnosed by preoperative puncture biopsy or postoperative pathological tissue examination at the Cancer Hospital, Chinese Academy of Medical Sciences.

### Cell culture and transfection

The HCCC-9810/HuCCT1/QBC939 cell line (a human hepato-cholangiocarcinoma cell line), was used to study the function of miR-380. HCCC-9810/HuCCT1/QBC939 cells were cultured in Dulbecco’s modified Eagle’s medium containing 10% fetal bovine serum, 100 U/ml penicillin and 0.1 mg/ml streptomycin, in an incubator containing 5% CO_2_ at 37℃. HCCC-9810/HuCCT1/QBC939 cells were inoculated into six-well plates 24 h before transfection, and were divided into eight groups: miR-NC, LIS1, miR-380, miR-380 + LIS1, In-NC(inhibitor), In-miR-380(inhibitor), si-LIS1(siRNA), and In-miR-380 + si-LIS1. The eight groups were transfected with miR-NC/In-NC, LIS1/si-LIS1 plasmid, miR-380 mimic/inhibitor, and cotransfected with miR-380 mimic/inhibitor and LIS1/si-LIS1. The cells were transfected using Lipofectamine 2000 (Invitrogen). A recombinant plasmid containing the target gene LIS1 3′-UTR was constructed to clone the primer (5′–3′) of LIS1 3′-UTR sequence in the genome of human cholangiocarcinoma cells. Upstream primer was: AAACTC-GAGTTGTGTCTCCTTCGGCCC (Containing *Xho*I restriction endonuclease site); and downstream primer was: AAAGCGGCCGCGGCATTTA-ATAGTTTACCAGTTGGT (containing *Not*I restriction endonuclease site). *Not*I and *Xho*I restriction endonucleases were obtained from Guangzhou Ruibo Biotechnology Co. Ltd. Transfected (plasmid or siRNA) si-LIS1 sequence (5′–3′) was: UGACCAUUAAACUAUGGGAUU; and control sequence (si-NC) was: AACGUACGCGGAAUACUUCGA. Transfection was performed when the cells reached 60–70% confluence.

### Detection of mRNA expression by qRT-PCR

TRIzol reagent was used to extract total RNA from cells and tissues. We used U6 as the internal reference for measurement of expression of miR-380, and GAPDH as the internal reference for measurement of mRNA expression of LIS1. Expression levels were measured by qRT-PCR. SYBR® PrimeScript ™ miRNA qRT-PCR Kit (Servicebio Corporation), G3326-15, Trizol (Servicebio Corporation, SCI1000-G), SweScript All-in-One First-Strand cDNA Synthesis SuperMix for qPCR (One-Step gDNA Remover) Servicebio Corporation, G3337-100). (Supplementary Table 1)

### Double-luciferase reporter gene assay to verify the regulatory effect of miR-380 on LIS1

We performed prediction using three miRNA databases including PITA, miRmap, and microT, and obtained the intersection of their results. The top 5 differentially expressed RNAs were selected for bioinformatics analysis using public databases. (Supplementary Table. Potential targets associated with mir-380) The TargetScan database predicts that miR-380 has a potential binding site on the 3′-UTR of LIS1. Wild-type and mutant luciferase reporter gene plasmids pMIR-WT-LIS1 and pMIR-Mut-LIS1 were constructed. The primer sequence of the mutant plasmid was: upstream 5′-GT-GAATCCAAATTGTATACTGTAAATTTACA-TACGTTGTCTAGA-3′; and downstream 5′-TCTAGA-CAACGTATGTAAATTTAGACCCTACAATT-TGGATTCAC-3′. The plasmids pMIR-WT-LIS1/pMIR-Mut-LIS1 were extracted and cotransfected with miR-380-3p mimic/inhibitor into HEK-293T cells. After 48 h transfection, the culture medium was discarded, the cells were washed with PBS, and cell lysate (20 µl) was added. The Luciferase activity of each group was detected using a Double-Luciferase Reporter Gene Detection kit. (Guangzhou Ruibo Biotechnology Co. Ltd.).

### Western blotting to detect protein expression

HCCC-9810/HuCCT1/QBC939 cell protein was extracted and the concentration was determined. Proteins were separated by 12% SDS-PAGE, then transferred to polyvinylidene difluoride membranes, and blocked with 5% skimmed milk at room temperature for 1 h. Primary LIS1 antibody (1:500), MMP-2 antibody (1:500), p-AKT antibody (1:500), AKT antibody (1:500), and GAPDH antibody (1:1,000; internal reference) were added to the polyvinylidene difluoride membranes and incubated at 4℃ overnight. Slides were washed and incubated with the secondary antibody (eroxidase-Conjugated Goat Anti-Rabbit IgG(H + L)) at room temperature for 1 h. Protein concentration was determined by enhanced chemiluminescence.

### Detection of LIS1 expression by immunofluorescence assay

The cells were inoculated into a culture dish with a treated cover glass. After the cells had nearly grown into a single layer, the cover glass was removed and washed twice with PBS. Logarithmic phase cells were centrifuged twice in PBS at 1000 rpm for 5 min, and cell smears were prepared. The cells were fixed in polyformaldehyde and sealed with a sealing solution for 30 min. LIS1 antibody diluted at 1:200 was added and the cells were incubated at room temperature for 1 h or 4℃ overnight. The cells were rinsed in PBST three times for 5 min each. Horseradish-peroxidase-labeled anti-rabbit secondary antibody (1:200) was added. The cells were incubated at room temperature in the dark for 1 h, rinsed in PBST three times for 5 min each, and once in distilled water. Fluorescence microscopy was used for observation.

### Detection of MMP-2 and p-AKT and LIS1 expression by immunohistochemistry

After graded dewaxing and hydration of paraffin sections, 3% H_2_O_2_ solution was added, and the sections were incubated at room temperature for 15 min to block endogenous peroxidase activity. The sections were placed in citrate buffer and subjected to microwave heating to repair the antigen. Add 5% normal sheep serum to seal and incubated at room temperature for 15 min. The following antibodies were added to the sections overnight at 4 ℃: LIS1 antibody diluted at 1:200, p-AKT antibody diluted at 1:500, and MMP-2 antibody diluted at 1:500. Rabbit secondary antibody labeled with horseradish peroxidase (1:200) was added, and the sections were incubated at 37℃ for 30 min. Horseradish-peroxidase-labeled *Streptomyces* ovalbumin working solution was dripped onto the sections, which were incubated at 37℃ for 30 min. After diaminobenzidene color development, the sections were stained with hematoxylin at room temperature for 2 min, followed by dehydration and neutral resin sealing. The sections were observed using an upright microscope.

### Cell counting kit 8 assay to detect cell proliferation

After 24 h of transfection, HCCC-9810/HuCCT1/QBC939 cells from the miR-NC, LIS1, miR-380, miR-380 + LIS1, In-NC(inhibitor), In-miR-380(inhibitor), si-LIS1(siRNA), and In-miR-380 + si-LIS1 groups were inoculated into 96-well plates with 2000 cells per well. Cell Counting Kit 8 (CCK-8) solution (10 µl) was added to each well, and the plates were incubated in the dark for 2 h. The optical density at 450 nm was measured with a microplate reader.

### Detection of cell cycle stage by flow cytometry

After transfection, HCCC-9810/HuCCT1/QBC939 cells in each group were digested with 0.01% trypsin for 30 min. The cells were resuspended with precooled PBS, and the cell suspension was added to precooled 70% ethanol for fixation. The cells were stored at 4℃ overnight. The ethanol was removed, cells were washed twice with PBS, 100 µl RNase A was added, and the suspension was incubated in water at 37℃ for 30 min. Next, 400 µl propidium iodide was added and the cells were incubated in the dark at 4℃ for 30 min. Flow cytometry was used to detect the cell cycle stage.

### Transwell assay for detection of cell invasiveness

After transfection, HCCC-9810 cells in each group were adjusted to a density of 1 × 10^6^/ml and placed in Matrigel overnight at 4℃. The next day, the Transwell chamber was precoated with Matrigel and 200 µl cell suspension was inoculated into the upper chamber. A total of 600 µl culture medium containing 10% fetal bovine serum was added to the lower chamber, and the cells were incubated at 37℃ for 24 h. The cells in the Transwell chamber were fixed in 95% ethanol for 10 min, stained with 0.5% crystal violet solution for 10 min, and washed with PBS. Cells in the upper layer of the filter membrane were wiped with a cotton swab, and cells in the lower layer of the filter membrane were observed under a light microscope.

### Statistical analysis

Data were expressed as mean ± standard deviation. The *t* test was used to compare data between two groups, and one-way ANOVA was used for comparison of data in three or more groups. SPSS version 22.0 was used for all statistical analysis. *P* < 0.05 was considered to indicate a significant difference.

## Results

### Expression of miR-380 in cholangiocarcinoma and its regulatory target gene

**Fig. 1:**
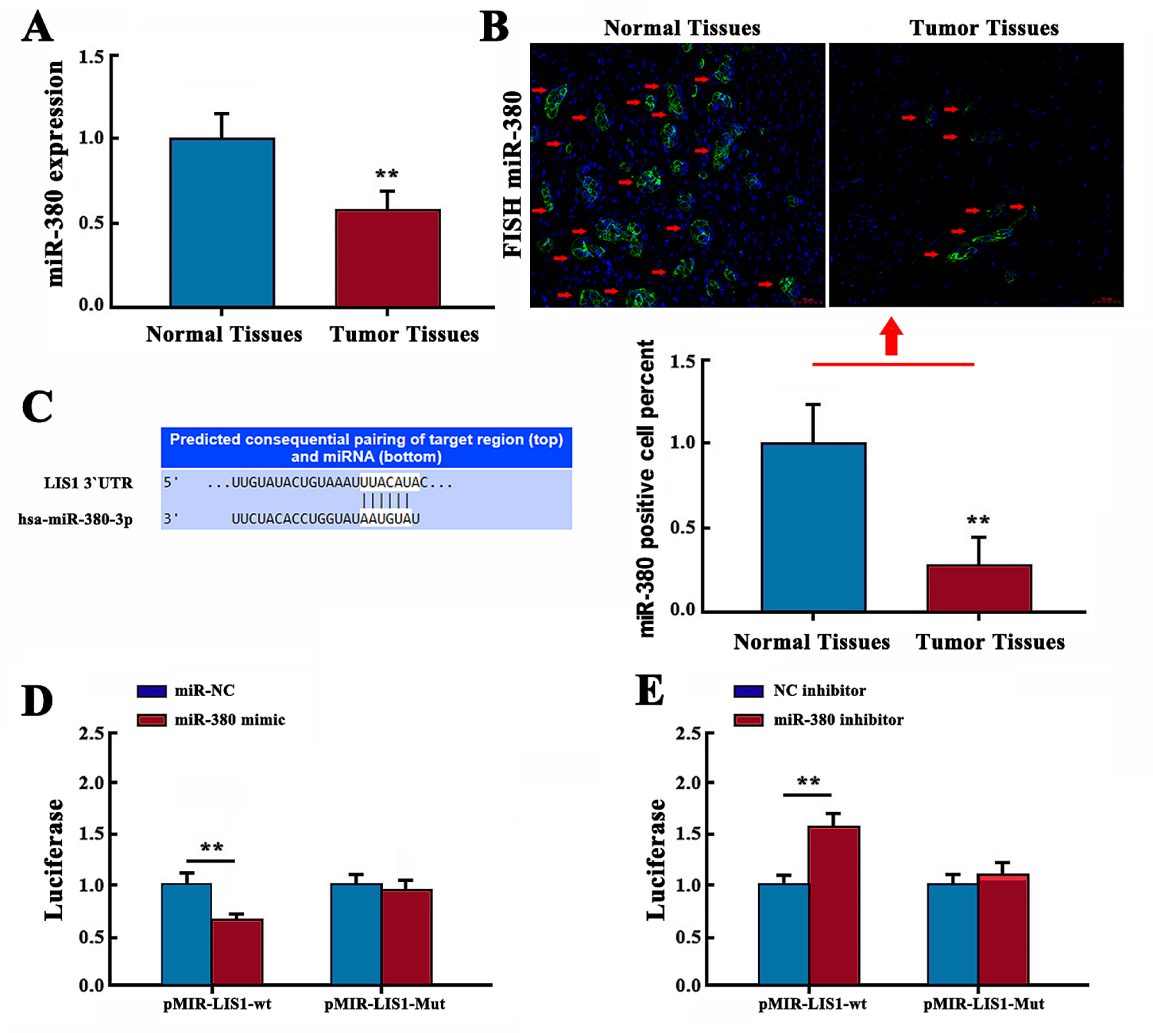
Expression and regulatory target gene of miR-380 in cholangiocarcinoma. **(A)** Expression of miR-380 in cholangiocarcinoma tissue was detected by qRT-PCR. **(B)** Percentage of miR-380-stained cells in cholangiocarcinoma tissue detected fluorescence in situ hybridization. Scale bar = 50 μm. **(C)** TargetScan database predicted the potential binding site of miR-380 on the 3′-UTR of LIS1. **(D, E)** Luciferase activity was detected by double-Luciferase reporter gene assay (**P < 0.01)

qRT-PCR showed that expression of miR-380 in cholangiocarcinoma tissue was significantly lower than in normal adjacent tissues (Fig. 1A) (*P* < 0.01). Fluorescence in situ hybridization found that the percentage of miR-380-positive cells in cholangiocarcinoma tissues was significantly lower than in normal paracancerous tissues (Fig. 1B), suggesting that expression of miR-380 may be related to cholangiocarcinoma.

The TargetScan database was used to screen for potential target genes of miR-380. There were potential binding sites on the 3′-UTR of LIS1 that were complementary to miR-380 (Fig. 1C). We constructed a LIS1 wild-type 3′-UTR fluorescein mei plasmid named pMIR- LIS1-wt. We also constructed a mutant plasmid, pMIR-LIS1-Mut, that predicted the 3′-UTR binding region and miR-380 binding region of LIS1. The primer sequence for constructing the mutant plasmid was as follows: upstream 5′-GT-GAATCCAATTGTATATGTAAATTTACA-TACGTGTCTAGA-3′; and downstream 5′-TCTAGA-CAACGTTATGTAAATTTACTACTACTACATTGGATTCAC-3′. HEK-293T cells were cotransfected with wild-type LIS1 3′-UTR reporter plasmid miR-380 (mimic/inhibitor). The luciferase activity assay showed that miR-380 mimic significantly decreased the activity of LIS1 3′-UTR (Fig. 1D) (*P* < 0.01). miR-380 inhibitor significantly increased the activity of LIS1 3′-UTR (Fig. 1E) (*P* < 0.01). There was no significant difference between the two groups for the mutation type LIS1 3′-UTR reporting vector (*P* > 0.05). After mutation of the binding site, miR-380 mimic/inhibitor still affected luciferase activity, but the extent of this effect was lower than that with the nonmutated groups. This indicated that the binding sites all affected the expression of luciferase activity, but the first binding site was more powerful. However, after mutation at all binding sites, miR-380 mimic/inhibitor did not affect the activity of luciferase.

### miR-380 negatively regulates LIS1 expression

**Fig. 2:**
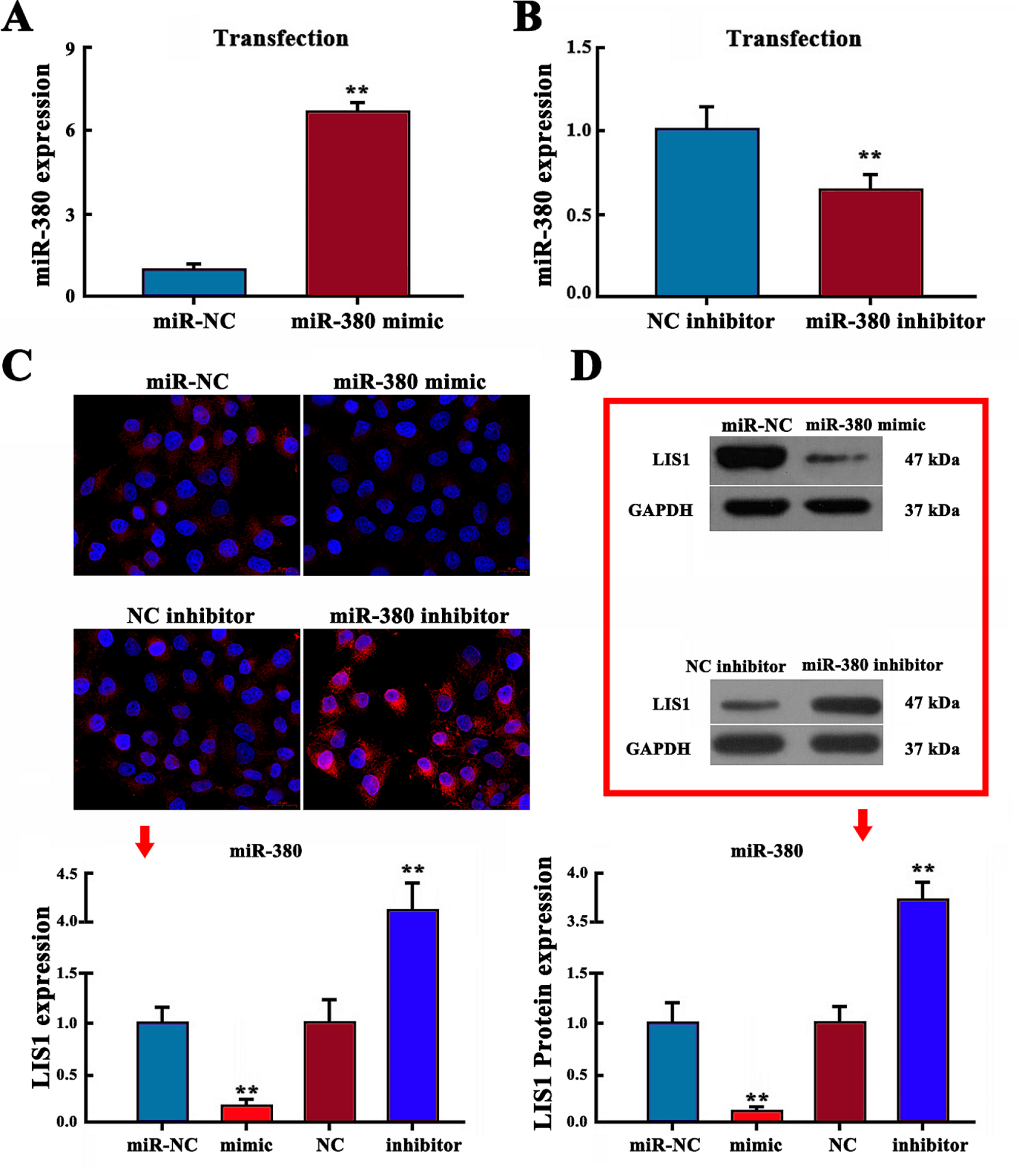
miR-380 negatively regulates LIS1 expression. **(A)** expression of miR-380 mimic was detected by qRT-PCR. **(B)** expression of miR-380 inhibitor was detected by qRT-PCR. **(C)** Immunofluorescence detection miR-380 mimic/inhibitor regulates the average fluorescence intensity of LIS1 protein. Scale bar = 20 μm. Western blotting was used to detect expression of LIS1 protein (**P < 0.01)

qRT-PCR showed that, after transfection of HCCC-9810 cells with miR-380 mimic, expression of miR-380 was significantly higher than in the control group, while the mRNA expression of LIS1 was significantly lower than in the control group (Fig. 2A, B). Immunofluorescence showed that miR-380 mimic decreased the average fluorescence intensity of LIS1 protein in HCCC-9810 cells, representing a decrease in expression (*P* < 0.05). The average fluorescence intensity of LIS1 protein in HCCC-9810 cells with miR-380 inhibitor was increased, and the expression level was significantly increased (Fig. 2C) (*P* < 0.01). Western blotting showed that miR-380 mimic significantly decreased expression of LIS1 protein in HCCC-9810 cells. In contrast, miR-380 inhibitor an increased the expression of LIS1 protein (Fig. 2D) (*P* < 0.01). These results indicate that miR-380 can negatively regulate its target gene, LIS1.

### Expression of LIS1 in cholangiocarcinoma

**Fig. 3:**
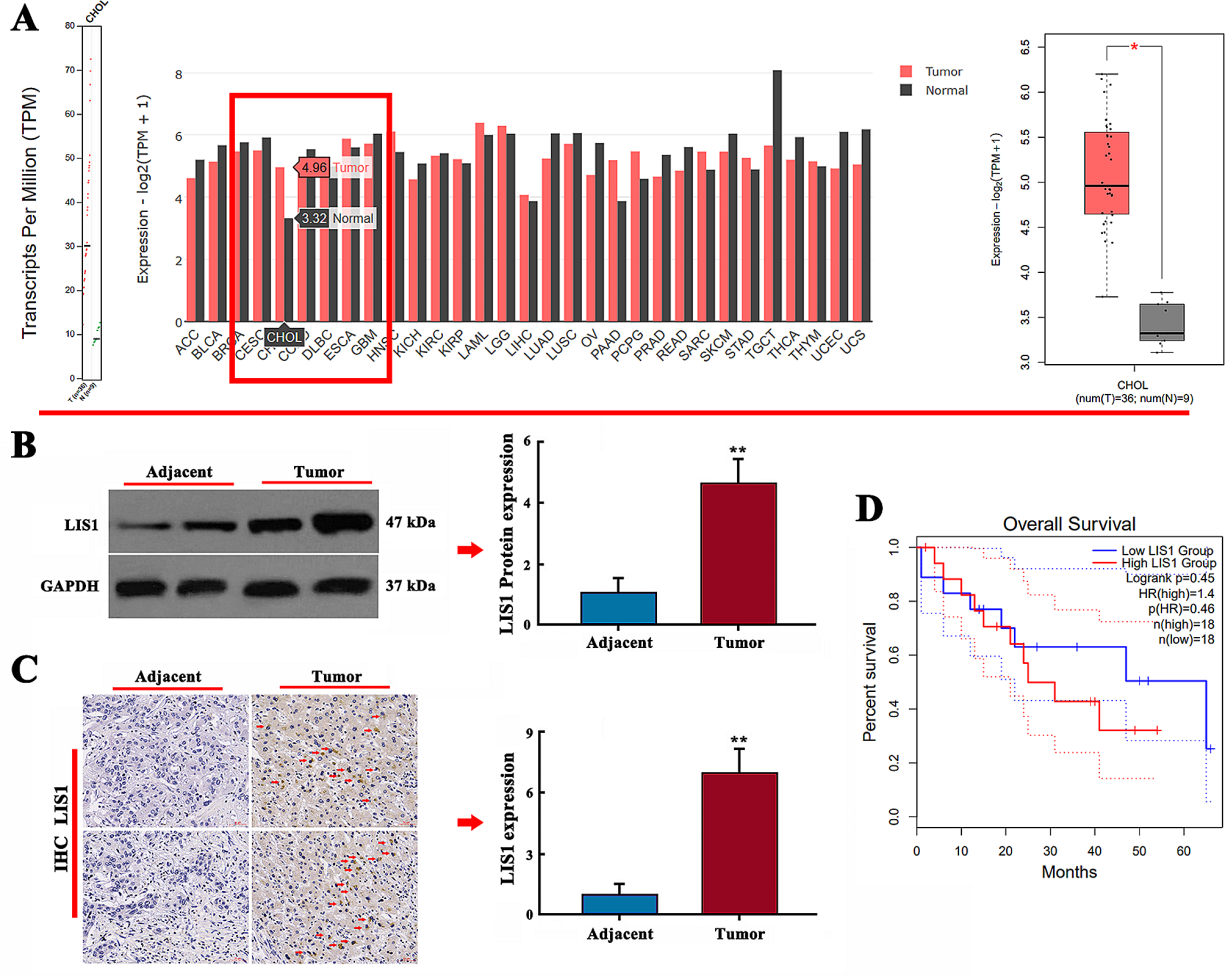
Expression of LIS1 in cholangiocarcinoma **(A)**. Expression of LIS1 in cholangiocarcinoma was predicted using GEPIA2, TCGA, and GTEx databases. **(B)** Western blotting detected expression of LIS1 protein. **(C)** Immunohistochemistry detected expression of LIS1 in cholangiocarcinoma and normal adjacent tissue. Scale bar = 20 μm. **(D)** Kaplan–Meier analysis and GEPIA2 database predicted that LIS1 was associated with survival in 18 patients with cholangiocarcinoma (**P < 0.01)

The GEPIA2 Health Information Database (http://gepia2.cancer-pku.cn/), TCGA (https://www.cancer.gov), and GTEx (https://gtexportal.org/) showed that LIS1 was highly expressed in cholangiocarcinoma. In 36 patients with cholangiocarcinoma and nine in the control group, LIS1 expression was significantly higher in the tissues of cholangiocarcinoma patients (Fig. 3A). Western blotting and immunohistochemistry showed that expression of LIS1 protein in cholangiocarcinoma tissue was significantly higher than in normal adjacent tissues (Fig. 3B, C). Kaplan–Meier analysis and GEPIA2 database found that the low survival rate of 18 patients with cholangiocarcinoma was positively correlated with high expression of LIS1. This suggests that expression of LIS1 is closely related to cholangiocarcinoma.

### Effects of miR-380 and LIS1 on the proliferation, cell cycle and invasiveness of HCCC-9810/HuCCT1/QBC939 cells

**Fig. 4:**
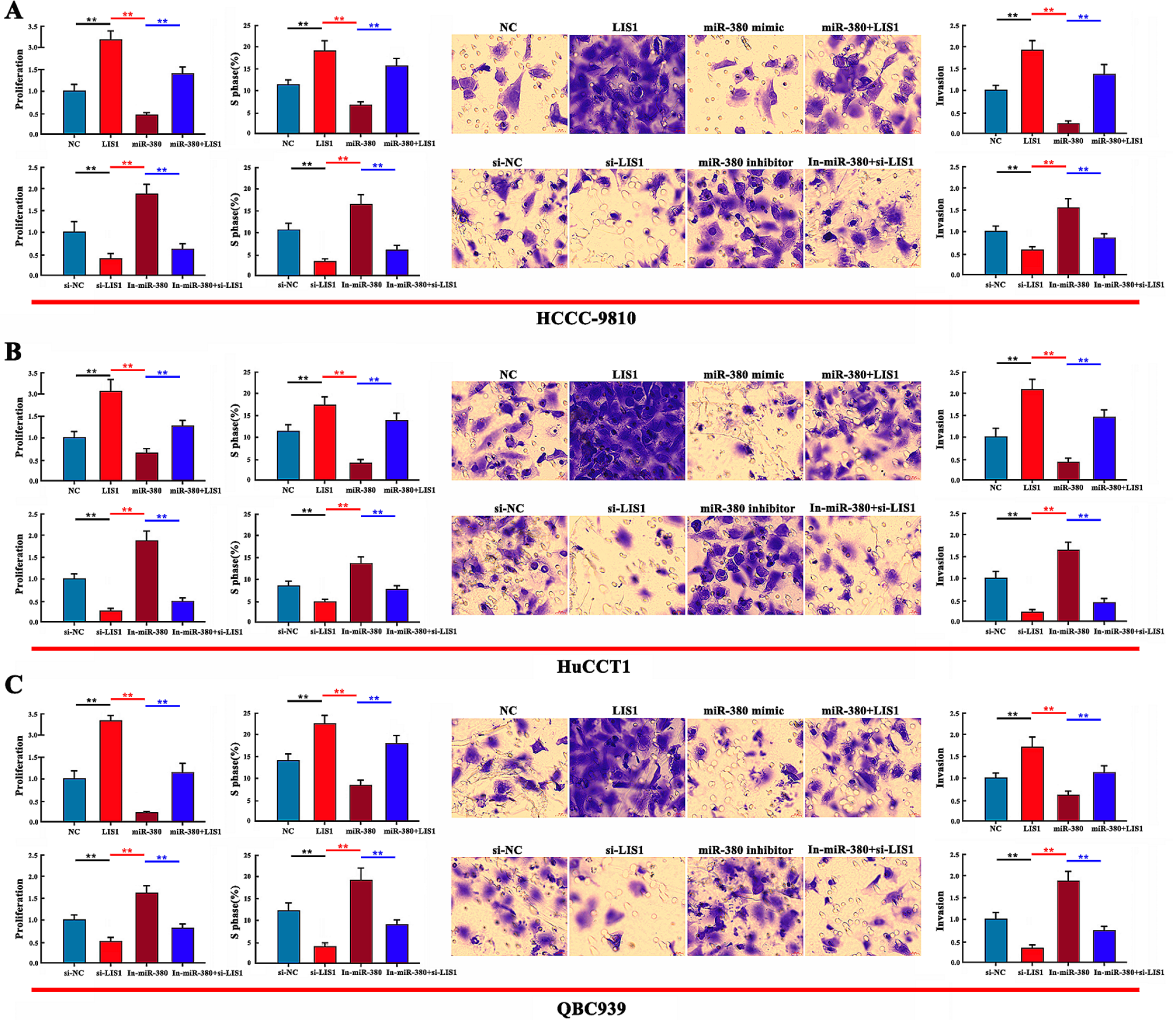
Effects of miR-380 mimic and LIS1 on proliferation, cell cycle, and invasiveness of HCCC-9810/HuCCT1/QBC939 cells. (**A**–**C**) CCK8 assay was used to detect proliferation of HCCC-9810/HuCCT1/QBC939 cells, and flow cytometry was used to detect the S-phase ratio. Cell invasiveness was detected by Transwell assay. Scale bar = 20 μm (**P < 0.01, *P < 0.05)

The regulation of proliferation, S-phase ratio and invasiveness of HCCC-9810/HuCCT1/QBC939 cells in the miR-380 mimic, In-miR-380(inhibitor), LIS1, si-LIS1(siRNA), miR-380 + LIS1 plasmid cotransfection, In-miR-380 + si-LIS1 plasmid cotransfection, and negative control groups was detected by CCK8, flow cytometry, and Transwell assay. Compared with the control group, the LIS1 and In-miR-380 groups significantly promoted cell proliferation, S-phase ratio and invasiveness of HCCC-9810/HuCCT1/QBC939 cells. The si-LIS1 and miR-380 mimic groups showed the opposite effect, inhibiting proliferation, S-phase ratio and invasiveness of HCCC-9810/HuCCT1/QBC939 cells. In the miR-380 + LIS1 plasmid cotransfection and In-miR-380 + si-LIS1 plasmid cotransfection group, LIS1 offset the inhibitory effect of miR-380 mimic. Compared with the miR-380 mimic group, the miR-380 + LIS1 plasmid cotransfection group significantly increased the proliferation, S-phase ratio and invasiveness of HCCC-9810/HuCCT1/QBC939 cells. si-LIS1 reversed the promotion of miR-380 inhibitor production. Compared with the miR-380 inhibitor group, the miR-380 + si-LIS1 plasmid cotransfection group significantly inhibited the proliferation, S-phase ratio and invasiveness of HCCC-9810/HuCCT1/QBC939 cells (Fig. 4A–C). These results indicate that miR-380 is involved in regulating the biological behavior of HCCC-9810/HuCCT1/QBC939 cells by negatively regulating LIS1.

### Effect of miR-380 mimic/inhibitor on expression of MMP-2, p-AKT and LIS1

**Fig. 5:**
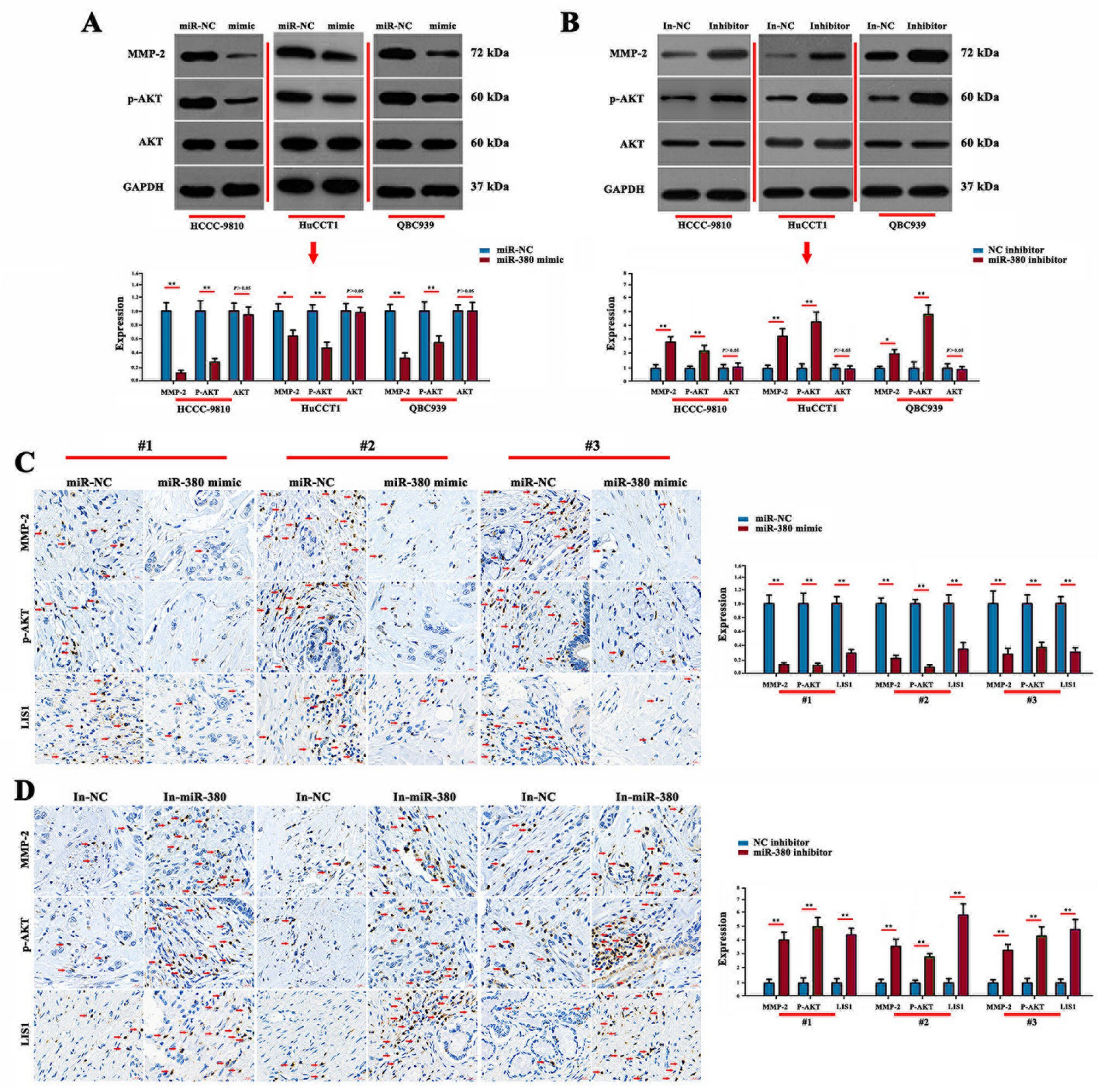
Western blotting and immunohistochemistry detected expression of MMP-2, p-AKT, and AKT. **(A)** Western blotting detected expression of MMP-2, p-AKT, and AKT protein by miR-380 mimic. **(B)** Western blotting detected the expression of MMP-2, p-AKT, and AKT protein in miR-380 inhibitor. **(C)** Immunohistochemistry detected the effect of expression of miR-380 mimic on MMP-2, p-AKT, and LIS1. Scale bar = 20 μm. **(D)** Immunohistochemistry detected the effect of expression of miR-380 inhibitor on MMP-2, p-AKT, and LIS1. Scale bar = 20 μm. (**P < 0.01, *P < 0.05)

Western blotting showed that expression of MMP-2 and p-AKT proteins in HCCC-9810/HuCCT1/QBC939 cells transfected with miR-380 mimic was significantly decreased. Expression of MMP-2 and p-AKT proteins in cells transfected with miR-380 inhibitor significantly increased, but the expression of AKT did not significantly change (Fig. 5A, B) (*P* > 0.05). Immunohistochemistry showed that miR-380 mimic significantly inhibited the expression of MMP-2, p-AKT and LIS1 (*P* < 0.01). miR-380 inhibitor significantly promoted expression of MMP-2, p-AKT and LIS1 (Fig. 5C, D) (*P* < 0.01). This suggests that miR-380 can inhibit the expression of MMP-,2 p-AKT and LIS1.

## Discussion

Studies have confirmed that the expression of specific miRNAs is closely related to the occurrence and pathological process of cholangiocarcinoma [30]. The results of our study showed that expression of miR-380 in cholangiocarcinoma tissue was significantly reduced. In vitro experiments showed that miR-380 affected expression of MMP-2 and p-AKT by targeting LIS1, thus participating in the regulation of the biological behavior of cholangiocarcinoma cells. In our study, qRT-PCR showed that expression of miR-380 was reduced in cholangiocarcinoma tissue, which suggests that the specific expression of miR-380 in cholangiocarcinoma tissue plays a role in tumor suppression. This is similar to previous studies. Many studies have shown that the expression of miR-380 is negatively correlated with the progression of various malignant tumors. Ling et al. reported that miR-380 inhibits the progression of prostate cancer by targeting neuronal cell adhesion molecule [31]. Ren et al. found that overexpression of miR-380 inhibits the proliferation and induces apoptosis of hepatocellular carcinoma cells [32]. Another study showed that miR-380 passed NF κ Pathway B targets MAP3K3 to inhibit the proliferation of non-small cell lung cancer cells [33].

It has been shown that miRNAs mainly cause target gene mRNA degradation or translation inhibition through complementary pairing with the 3′-UTR base of the target gene mRNA; thus, regulating target gene expression at the post-transcriptional level. Identification of miRNA target genes is an important method for determining the roles and mechanisms of specific miRNAs [34]. In our study, screening of the TargetScan database indicated that LIS1 was a potential target of miR-380. Subsequent experiments indicated that miR-380 specifically bound to the 3′-UTR of LIS1. Transfection of HCCC-9810 cells with miR-380 mimic indicated that upregulation of miR-380 inhibited expression of LIS1 protein. At the same time, after miR-380 mimic treatment, the average fluorescence intensity of LIS1 protein in HCCC-9810 cells decreased. It was also found that miR-380 inhibitor upregulated the expression and average fluorescence intensity of LIS1 protein, which confirmed that LIS1 was negatively regulated by miR-380.

Immunohistochemistry and western blotting showed that there was abnormally high expression of LIS1 in cholangiocarcinoma tissue, suggesting that increased expression of LIS1 may be closely related to cholangiocarcinoma. This is similar to the results of previous studies in which LIS1 played an important role in the development of liver cancer, lung cancer, and other malignancies [35, 36]. To explore further the molecular mechanism of miR-380 and its target gene in cholangiocarcinoma, we studied the regulatory effect of miR-380 and LIS1 on cholangiocarcinoma cells. After HCCC-9810/HuCCT1/QBC939 cells were transfected with miR-380 mimic, cell proliferation, S-phase arrest and invasiveness were significantly reduced. At the same time, we confirmed that miR-380 inhibitor had the opposite effects to miR-380 mimic. In contrast, upregulation of LIS1 expression promoted the malignant behavior of the cells and reversed the regulatory effect of miR-380 mimic, and knocking out LIS1 preserved the promotive effect of miR-380 inhibitor. In vitro experiments showed that miR-380 negatively regulated LIS1 to inhibit the malignant behavior of cholangiocarcinoma cells.

Downregulation of LIS1 is related to activation of the PI3K/AKT pathway, thus promoting the occurrence and development of tumors [37]. Abnormal activation of the PI3K/AKT signaling pathway is related to a variety of biological processes, such as cell proliferation, growth, differentiation and apoptosis, and thus plays an important role in cancer. In our study, western blotting showed that upregulation of miR-380 inhibited the expression of MMP-2 and p-AKT; the main proteins of the PI3K/AKT signaling pathway. We also verified that miR-380 inhibitor promoted the expression of MMP-2 and p-AKT proteins. mmunohistochemistry showed that miR-380 mimic inhibited the expression of MMP-2, p-AKT and LIS1, and that miR-380 inhibitor had the opposite effect. However, we did not further explore whether miR-380 promotes the degradation or translation inhibition of LIS1. We will also describe this limitation of our study in the discussion. Previous research has demonstrated that miR-380 can promote the pathogenesis of neuroblastoma by degrading p53. Based on this, we hypothesize that LIS1 may be degraded in a similar manner. These results suggest that miR-380 affects the expression of MMP-2 and p-AKT through targeted regulation of LIS1, thus participating in the regulation of cholangiocarcinoma cells, and confirm that miR-380 has an antitumor effect on cholangiocarcinoma.

## Conclusions

Expression of miR-380 is abnormal in cholangiocarcinoma, and its upregulation reduces the expression of MMP-2/p-AKT through targeted regulation of LIS1, thereby inhibiting the proliferation, S-phase arrest and invasiveness of cholangiocarcinoma cells. Our results indicate that miR-380 has an antitumor effect on cholangiocarcinoma.

### Electronic supplementary material

Below is the link to the electronic supplementary material.

Supplementary Material 1

Supplementary Material 2

## Data Availability

The data that support the findings of this study are available from the corresponding author, Xinyu Bi, upon reasonable request.
