# Supplementary material for: MiR-380 inhibits the proliferation and invasion of cholangiocarcinoma cells by silencing LIS1
Source: Cancer Cell Int. 2024 Apr 6;24:129. doi: 10.1186/s12935-024-03241-4 (PMC10998336; doi:10.1186/s12935-024-03241-4)
Supplement: Supplementary file 1 — Supplementary Material 1 [file 12935_2024_3241_MOESM1_ESM.docx]

**Supplementary table 1.**PCR primer sequences

| Gene | Primer sequence |
| --- | --- |
| miR-380-3p | F：5'-ACACTCCAGCTGGGTATGTAATGTGGTCC-3'  R：5'-TGGTGTCGTGGAGTCG -3' |
| U6 | F：5'-ATTGGAACGATACAGAGAAGATT-3'  R：5'-GGAACGCTTCACGAATTTG-3' |
| LIS1 | F：5'-CGTCTCTGGTCACCTGTGTAA-3'  R：5'-CTCATGGCTGTCCGTCGAT-3' |
| GAPDH | F：5'-TGTTCGTCATGGGTGTGAAC-3'  R：5'-ATGGCATGGACTGTGGTCAT-3' |
